# The prevalence of risk factors for foot ulceration in patients with end stage renal disease on haemodialysis

**DOI:** 10.1186/1757-1146-4-S1-O22

**Published:** 2011-05-20

**Authors:** Michelle Kaminski, Nicoletta Frescos, Stephen Tucker

**Affiliations:** 1 Podiatry Department, Eastern Health, Melbourne, Victoria, 3152, Australia; 2 Podiatry Department, La Trobe University, Melbourne, Victoria, 3152, Australia

## Background

End stage renal disease (ESRD) has been associated with foot ulceration and lower extremity amputation (LEA). However, the underlying risk factors for foot ulceration have received limited attention in this population. The aim of this study was to investigate the prevalence and type of risk factors for foot ulceration present in patients with ESRD on haemodialysis without the coexistence of diabetes mellitus (DM).

## Methods

One hundred and ninety participants with ESRD and/or DM were recruited over a six week period. Participants were allocated into one of three groups: (i) ESRD without DM, (ii) DM without ESRD and (iii) coexisting ESRD and DM. Participants were screened for the risk factors for foot ulceration. Statistical comparisons were made between the three groups for both the prevalence and type of risk factors using a Fisher’s Exact Test.

## Results

Risk factors for foot ulceration were found to be highly prevalent in the ESRD population. Participants with both ESRD and DM exhibited statistically significant differences in risk factor presentation for peripheral neuropathy (p=0.033), vascular insufficiency (p=0.001) and footwear (p=0.037) in comparison to participants with DM alone.

## Conclusions

There are high prevalence rates of risk factors for foot ulceration in the ESRD population on haemodialysis and are comparable to those with DM. Individuals with coexisting ESRD and DM have an even greater risk for foot ulceration and LEA. This highlights the importance that regular foot screening, preventative education and treatment are necessary for patients with ESRD to potentially reduce the risk of foot ulcerations and LEAs.

